# Design, Implementation and Experimental Investigation of a Pedestrian Street Crossing Assistance System Based on Visible Light Communications

**DOI:** 10.3390/s22155481

**Published:** 2022-07-22

**Authors:** Alin-Mihai Căilean, Cătălin Beguni, Sebastian-Andrei Avătămăniței, Mihai Dimian, Valentin Popa

**Affiliations:** 1 Integrated Center for Research, Development and Innovation in Advanced Materials, Nanotechnologies, and Distributed Systems for Fabrication and Control, Stefan cel Mare University of Suceava, 720229 Suceava, Romania; catalin.beguni@usm.ro (C.B.); sebastian.avatamanitei@usm.ro (S.-A.A.); dimian@usm.ro (M.D.); 2 Department of Computers, Electronics and Automation, Stefan cel Mare University of Suceava, 720229 Suceava, Romania; valentin@eed.usv.ro; 3 Laboratoire D’ingénierie des Systèmes de Versailles (LISV), Paris-Saclay University, 78140 Velizy-Villacoublay, France

**Keywords:** accident prevention, I2V, optical communications, presence sensors, street crossing, traffic safety, vehicle safety applications, visible light communications

## Abstract

In urban areas, pedestrians are the road users category that is the most exposed to road accident fatalities. In this context, the present article proposes a totally new architecture, which aims to increase the safety of pedestrians on the crosswalk. The first component of the design is a pedestrian detection system, which identifies the user’s presence in the region of the crosswalk and determines the future street crossing action possibility or the presence of a pedestrian engaged in street crossing. The second component of the system is the visible light communications part, which is used to transmit this information toward the approaching vehicles. The proposed architecture has been implemented at a regular scale and experimentally evaluated in outdoor conditions. The experimental results showed a 100% overall pedestrian detection rate. On the other hand, the VLC system showed a communication distance between 5 and 40 m when using a standard LED light crosswalk sign as a VLC emitter, while maintaining a bit error ratio between 10^−7^ and 10^−5^. These results demonstrate the fact that the VLC technology is now able to be used in real applications, making the transition from a high potential technology to a confirmed technology. As far as we know, this is the first article presenting such a pedestrian street crossing assistance system.

## 1. Introduction

Visible light communications (VLC) are a relatively new wireless communications technology in which the information is modulated onto the optical carrier of visible light [1,2,3]. This unique feature makes VLC a dual-purpose technology that can simultaneously support lighting and data communication. Moreover, unlike any other wireless communications technologies, the data broadcasting function is enabled without extra energy consumption for data carrier generation. So, VLC is developed based on the ubiquitous character and the fast-switching ability of solid-state lighting (SSL) sources such as light emitting diodes (LEDs) [4,5,6,7]. As well known, SSL light sources are extremely energy-efficient, benefit from an extended lifetime that can reach up to 50,000 h, and have switching times that tend to go down to ns values [6,7], enabling in turn very high data rates [8]. Thus, the VLC technology has the potential to transform any LED light source into a data broadcasting unit [1,2,3,6,7]. On the receiver side, VLC assumes the direct detection of the incident light with the help of fast response time optical detectors, usually positive intrinsic negative (PIN) photodiodes with nanoseconds response times. In order to comply with the eye safety norms and with the lighting standards, the data transfer is considered a complementary function, in addition to lighting, and therefore, the communication function should not affect in any way the primary purpose of the device [9]. Consequently, VLC must not introduce any visible flickering, and it should enable light dimming if the user requires it. These aspects have been stipulated in the IEEE 802.15.7 standard for short-range optical communications using visible light [9]. In indoor applications, the VLC technology has reached a relatively high maturity level, with laboratory prototypes being able to provide tens of gigabits per second data rates [10,11,12] and with commercially available systems being able to provide up to 1 Gb/s internet connections [13].

On the other hand, due to numerous challenges associated with the unpredictable vehicular communication channel [14,15,16], the development of automotive VLC systems has remained several steps behind compared to indoor VLC solutions. It should be mentioned here that the use of the VLC technology in vehicular applications was considered by some rather debatable, due to the numerous external factors, which raised reliability uncertainties. The most important perturbing factors are on the one hand the multitude of optical noise factors that interfere with the data signal, and on the other hand, the weather phenomena that interfere with the light passage [14,15,16,17,18,19,20]. So, as shown in [17,18], water particles from rain and fog affect light passage through a combination of reflection and refraction, whereas heavy dust [19] and snowfall [20,21,22] affect VLC performances as they block light from reaching the photosensitive element’s surface. Nevertheless, in the last few years, recent achievements in the area of automotive VLC systems have made this relatively new technology [14,16] more advanced and reliable than ever. Thus, recent years have shown vehicular VLC prototypes that are able to provide performances that were inconceivable a decade ago. In terms of noise resilience, some of the current prototypes are able to keep an active link even in strong sunlight exposure [21,23]. In terms of communication ranges, the maximum reported distance has reached 180–188 m in outdoor conditions, while providing data rates of up to 100 kb/s [24,25]. In terms of mobility, it has been shown that with proper optical systems, VLC prototypes are able to cover wide angles and to provide improved connectivity [26,27]. Furthermore, vehicular VLC systems are able to provide all these benefits while also being able to deliver very low latencies [23,28,29], whereas if the application requires it, these systems are also capable of providing light dimming capabilities [30]. All these recent achievements have confirmed a high potential associated with the use of the VLC technology in communication-based vehicle safety applications. Nevertheless, in order to move forward, additional work is required concerning the experimental evaluation of vehicular VLC prototypes in scenarios as close as possible to real-life applications. These field tests should demonstrate that the VLC technology is able to be useful in realistic situations.

In this context, this article presents the aspects related to the design, implementation, and experimental evaluation of a VLC system integrated into a real-life application. The proposed system is intended to provide intelligent or autonomous vehicles with support in dealing with zebra crossing areas. Thus, the proposed system is able to monitor a pedestrian crossing, evaluate if a person is in that area, and signal whether or not, any person is on the way to crossing the street. The result of this assessment is translated into a six-level alert state, with level 1 indicating that no one is near the pedestrian crossing, and level 6 indicating that for sure, a person is on the way to crossing the street. Next, this information is transmitted to the approaching vehicles using the VLC technology. In this case, the VLC system consists of an on-vehicle VLC receiver, and an LED emitter developed based on a street crossing sign equipped with standard power orange signaling lamps. The experimental results show an overall 100% pedestrian detection rate and a communication distance between 5 and 40 m. As far as we know, this is one of the first articles that report an experimental investigation concerning the performances of VLC systems in such an application. Additionally, as the experimental investigation has shown, the proposed system has improved performance compared with other solutions, providing a very high pedestrian detection rate at the crosswalk level, and a high capacity for transmitting this information toward approaching vehicles. An important advantage of the proposed concept comes from the fact that this method assumes pedestrian detection at the crosswalk level. As pedestrians have relatively low speed, it is clear that a pedestrian detection system located at the crosswalk level will have sufficient time to detect any potential passer-by. On the other hand, a speeding car approaching a crosswalk has just very few seconds to detect the pedestrian. Moreover, on-vehicle detection systems, (i.e., on-vehicle camera systems, on-vehicle radar, or LIDAR systems) can definitely encounter pedestrian detection problems in scenarios in which visibility is blocked by different obstacles, (i.e., other cars, or different objects located in the crosswalk area such as trees). On the other hand, once a pedestrian is detected, the VLC technology enables this information transmission in times that are below 20 ms [23,28,29].

The rest of this article is structured as follows. Section 2 presents the motivation of this work, showing the high impact road accidents have on human life and on human society, while also pointing out the importance of new technologies focused on improving the safety of the most vulnerable road users category, namely pedestrians. Section 3 debates the aspects related to the design and the implementation of the proposed prototype. Section 4 describes the experimental evaluation process applied to the proposed road safety architecture and delivers the experimental results confirming the benefits of the proposed prototype. Section 5 provides a debate concerning the benefits of the proposed road safety architecture and a comparison with other solutions envisioned for pedestrian presence applications. In the end, Section 6 delivers the conclusions of this work.

## 2. Road Accidents in Figures: The Vulnerability of Pedestrians and Solutions to This Issue

Up until the COVID-19 pandemic, road accidents were one of the top ten causes of death worldwide, with an average world mortality rate reaching up to 169 deaths per million inhabitants [31,32,33,34]. In the pandemic context, one can estimate that the number of road fatalities decreased from an average of 1.2–1.35 million per year in the 2015–2019 [31,32,33,34], to an estimated number of fatalities of 1.1 million in 2020 and 1.15 million in 2021. The reasons behind this fatality rate improvement are mainly given by the mobility limitations imposed by the pandemic situation. So, under the total lockdown conditions, there were countries where the road fatality rate decreased by 80% (i.e., the case of New Zealand in April 2020 compared to April 2019). In the European Union, the road fatalities number decreased by 17% in 2020 compared to 2019 [35]. On the other hand, in 2020, the total number of fatalities has increased by 7.2% in the USA, although the pandemic conditions reduced driving by 13.2% [36]. This last statistic demonstrates that less driving does not necessarily equals fewer road fatalities and that significant efforts are required to further improve road safety.

Nevertheless, as the pandemic is coming to an end, and human life is returning to normal habits, the increased mobility is most likely to generate a surge in the number of road victims. As a matter of fact, it is expected that, in the return to normality, the number of victims will probably be 3–5% higher than before the pandemic, followed by a decrease to pre-pandemic figures after 2–3 years. This increase will be caused by a lack of mobility in the last period, which affected the driving experience, and which will most probably lead to more distracted driving habits. The mortality rate related to road accidents will most probably be affected by psychological factors associated with isolation, media exposure, and post-pandemic stress disorders [37,38]. All these causes will affect all road users’ attention, including pedestrians. 

Now, if one examines the road fatality figures, one can see that the European countries have the lowest mortality rate due to road accidents. According to official data [39], in 2019 this average rate was about 51 deaths per million inhabitants in the European Union (EU) member countries, varying from 22 in Sweden to 96 in Romania. Moreover, if it is to further zoom into the road fatalities statistics, one can see that in EU countries, about 44% of the road victims were car drivers or car passengers, 20.2% of the victims were pedestrians, 18% motorcyclists and moped users, whereas 9% were bikers. In Romania, the pedestrian mortality rate is by far the highest among the EU countries, reaching 39.1%, which is almost double the average rate. As mentioned above, Romania is also the EU country with the highest mortality rate due to road accidents. In 2016–2019, this rate was 96 to 97 deaths per million inhabitants. Less proper road infrastructure, road signaling deficiencies, and a large road network passing through inhabited areas can be considered as the main reasons behind this higher mortality rate.

**Figure 1:**
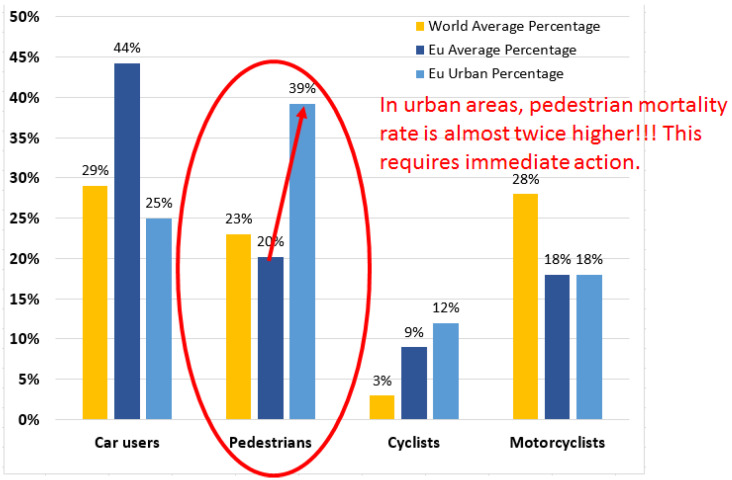
Percentage distribution of the mortality among the main road user categories, suggesting the high vulnerability rate among pedestrians, especially in urban areas.

Now, if it is to return to EU road fatalities statistics and move our attention from the overall figures to the urban area figures, one can see that vulnerable road users represent 70% of road fatalities, with pedestrians representing about 40% of the fatalities (see Figure 1). Thus, these figures show that even if modern society is struggling to improve human life in urban areas, these areas are significantly more exposed for the vulnerable road users categories, (i.e., pedestrians, motorcyclists, and bikers). The vulnerability of pedestrians to road accidents is also amplified by the distraction caused by mobile phone use during walking [40,41,42].

In summary, one can see that in EU countries, and not only, pedestrians are the most exposed road user category in urban areas. To ameliorate this situation, the improvement of vehicles, road infrastructures, and the integration of smart technologies into road safety applications are the most concrete solutions. These solutions are fully compatible with the current smart cities trend and envision the integration of state-of-the-art sensors and wireless communication technology in order to improve the safety of vulnerable road users. Consequently, one can see that the architecture proposed in this article is addressing these challenges, as it proposes a smart pedestrian crosswalk, that aims to improve the safety of road traffic by increasing the awareness and the ability to detect pedestrians, broadcasting this information toward the approaching vehicles with the help of state-of-the-art wireless communication technology.

## 3. Design and Implementation of the Pedestrian Crosswalk Assistance System

**Figure 2:**
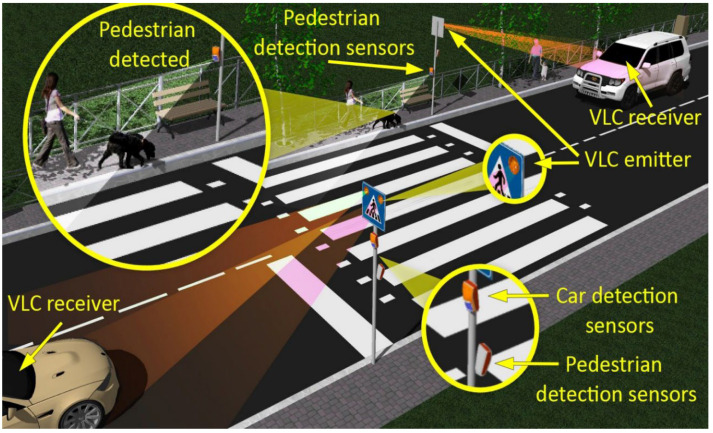
Functionality of the envisioned architecture: based on localization sensors, the intelligent crosswalk is able to identify pedestrians that might use the crosswalk; these data are then transmitted by the LED traffic sign toward the approaching vehicles by using the visible light communications technology.

In the context mentioned above, this article proposes an architecture designed to increase the safety of pedestrians using a crosswalk. The proposed architecture consists of two main components. The first one is a pedestrian presence and localization unit that has the purpose to detect a person intending to use the crosswalk. Next, after the detection of a person by the embedded sensors, a software algorithm is used to establish the eventual intention in street crossing, based on a sensor’s data fusion process. The second component of the architecture consists of a VLC system that is used to transmit information concerning any eventual street crossing intention toward the approaching vehicles. The functionality of the envisioned architecture is illustrated in Figure 2.

### 3.1. Pedestrian Presence and Localization Unit

**Figure 3:**
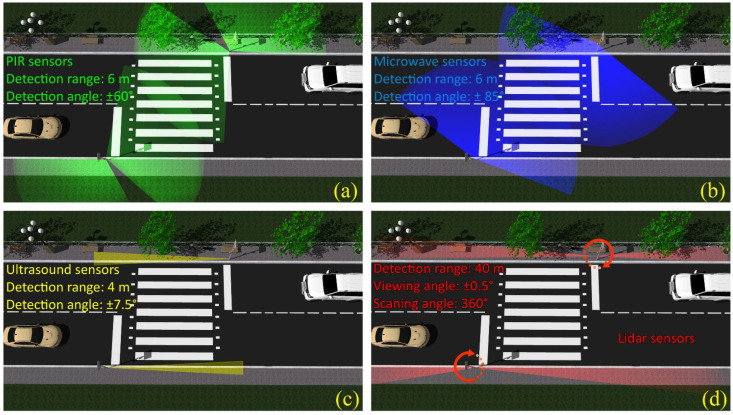
Schematic of the pedestrian presence and localization unit sensors and their coverage area information: (**a**) PIR sensors; (**b**) microwave sensors; (**c**) ultrasound sensors; (**d**) LIDAR sensors.

This section presents the aspects related to the implementation of the pedestrian detection and localization system. As shown in Figure 2 and Figure 3, this system contains several types of presence and localization sensors, whose activities are driven by a 180 MHz ARM Cortex M4 microcontroller board using a dedicated software component. 

So, one of the main goals of this experiment is to determine which sensors are most suitable for this task and what are the obtainable results in the end. Passive infra-red (PIR) sensors are perhaps the most popular proximity sensors used to identify human movement. This type of sensor detects a living creature’s presence and movement by capturing and focusing the type C infrared radiation (3 µm–1 mm). As humans emit thermal radiation in the micrometer domain, these sensors enable their accurate detection. In direct relation to this radiation, the value of the currents generated through the sensor is compared to determine the variations, allowing the trigger to certify the movement. The capturing and focusing of the infrared radiation are performed through a series of Fresnel lenses, most often embedded in a spherical shape, which have the optical sensor as a focal point. The position of the infrared radiation source(s), (i.e., human or animal) with respect to the position of the incident surface, allows the electronics to determine the motion by simply analyzing the value of the signals generated by the sensor. From a constructive point of view, the modules based on PIR sensors allow the adjustment of the sensitivity and of the delay times. Once the sensor detects human or animal movement, its output value becomes a logical “*1*”, which is maintained for a certain time. Compared to other proximity sensors, PIR sensors have an obvious advantage given their ability to exclusively identify humans and, in some cases, large animals’ movement. The movement of all other objects that are not able to generate a C-type IR radiation will never generate false detections. As a disadvantage, these sensors may experience difficulties on hot summer days, when the ambient temperature within the sensor’s detection range is close to 37° Celsius. So, in such conditions, the functionality of PIR sensors might be affected, as these sensors are mostly suitable for indoor applications or for scenarios where sunlight interferences are limited [42,43,44]. Additional information concerning the use of PIR sensors in human detecting applications can be found in [42,43,44]. In the proposed architecture, the PIR sensors have been initially envisioned to detect pedestrians that are on the sidewalk within the crosswalk region and pedestrians engaged in street crossing (see Figure 3a).

The microwave sensors are known for their theoretical high sensitivity and high resolution. Microwave proximity sensors detect motion using the Doppler effect [45,46,47]. Such a sensor constantly emits a microwave electromagnetic signal whose frequency shifts in accordance with the moving surfaces it encounters. The reception of the resulting frequency indicates the presence of a moving object within the perimeter of interest. Compared to the PIR sensor, microwave sensors are recognized as having better overall performance, as they are more sensitive and precise. Additionally, this type of sensor is not influenced by temperature. Microwave sensors have a rather similar maximum detection distance as the PIR sensors, (i.e., approx. 6 m), but have a disadvantage in the fact that they detect any movement, not just pedestrians’ movement. In addition, their placement near metal objects can lead to their invalidation or malfunction. Additionally, the performances of such sensors can be affected by a high degree of humidity (associated with thick fog for example, especially in applications that envision long detection distances, (i.e., 4–6 m). Figure 3b. illustrate the microwave sensors envisioned positioning and their coverage area in the preliminary test. Thus, the microwave sensors could be used as complementary devices for PIR sensors, covering each other’s disadvantages, and enabling improved pedestrian detection performances [45,46,47].

Ultrasonic proximity sensors have a similar operating principle as microwave sensors. In order to determine the distance between the sensor and the surrounding objects, our selected sensors emit a 20–40 kHz wave signal, having a beam angle of ±7.5 degrees. The emitted wave is reflected by the surrounding objects, and the reception of its echo allows the determination of the distance with an accuracy of up to 3 mm. The output of the ultrasonic device offers a TTL signal in relation to the distance between the sensor and the objects found in the FOV. Although the maximum measurable distance is limited to 4 m, the high accuracy is advantageous in certain situations compared to the sensors mentioned above. Although the speed of sound in the air may be influenced by temperature and humidity, the accuracy of such sensors remains satisfactory. Similar to microwave sensors, they are unable to exclusively determine human movement, generating information about any form of movement in relation to their position. Additionally, a possible disadvantage of ultrasonic proximity sensors could be given by the fact that some pets, (e.g., dogs and cats) are able to perceive ultrasounds, and might be perturbed by these sensors. Additional details concerning the integration of ultrasonic sensors in automotive applications can be found in [48], where the performances of these sensors are compared to the performances of other localization sensors used in commercial vehicles. Due to the narrow FOV, the ultrasonic sensors have been chosen to determine the moment/point when the pedestrian is on the way to leaving the sidewalk and stepping on the crosswalk (see Figure 3c). 

The LIDAR sensor has been included in the test due to its high precision and long detection range [48,49,50,51,52]. Based on these unique features, LIDAR sensors are widely used in automotive applications, being one of the most advanced solutions used for object identification and localization [48,49,50,51,52]. The LIDAR V3 sensor has a range of up to 40 m while providing a centimeter accuracy, (i.e., less than ±10 cm). Compared to other LIDAR sensors, this one has a relatively lower cost and rather inferior performance. The LIDAR V3 sensor has a FOV of ±0.5°, requiring a rather precise positioning with respect to the object to be detected. Therefore, in order to extend the angular detection area, the LIDAR has been mounted on an angular controlled servo motor, enabling the system to cover the area of the sidewalk and the area of the crosswalk, having this way a 360-degree scanning area. Similar to microwave and ultrasonic proximity sensors, LIDAR sensors emit a modulated electromagnetic signal, (i.e., this time a 905 nm optical signal), whose reflected radiation allows the sensor to determine the distance to the object based on a time-of-flight analysis. The performances of the LIDAR sensor might be influenced by direct exposure to strong sunlight, whereas snowfall or dense fog might determine false detections. This sensor can be used to sense persons approaching the crosswalk, to determine when a person is on the way to crossing the street, and to establish that someone is on the crosswalk. The information from this sensor is used to determine that a person is present in the area of interest and also to provide information concerning the pedestrians’ location (see Figure 3d).

**Figure 4:**
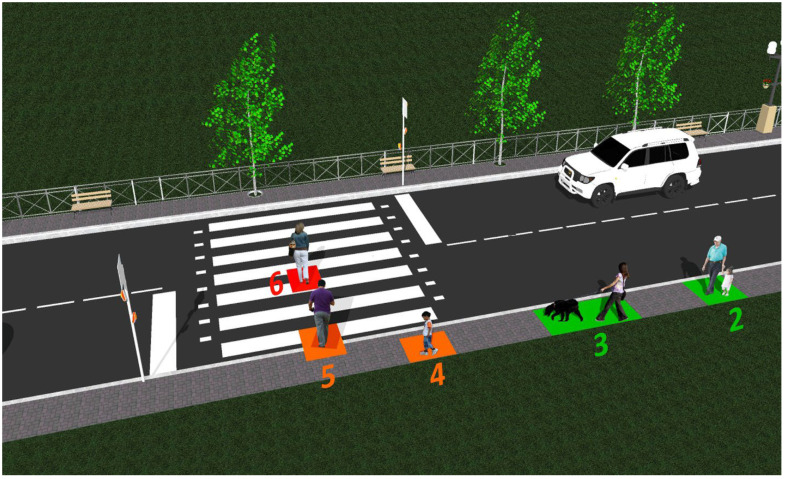
Illustration of the areas associated with the six alert levels. The color of the region indicates the danger level associated with each zone: green—low danger zone, orange—medium danger zone, red—high danger zone.

In addition to the devices described above and whose performances are summarized in Table 1, the pedestrian presence and localization unit contains a 180 MHz ARM Cortex M4 microcontroller that monitors the information received from the sensors. Based on data analysis, and based on a software algorithm, the embedded microcontroller is able to make an assessment concerning the alert level. Figure 4 illustrates the six alert levels determined by the presence of a pedestrian in a certain location. For the case when sensors detect multiple persons, the highest alert level will be considered. More exactly, the microcontroller will evaluate the information received from the presence and localization sensors and will decide from one of the alert levels. The software algorithm has been developed based on a safety-first approach, and so, a false positive message is preferred to a false negative. In other words, it is preferable to transmit a false alert warning message than to miss an event.

### 3.2. Visible Light Communications Component

**Figure 5:**
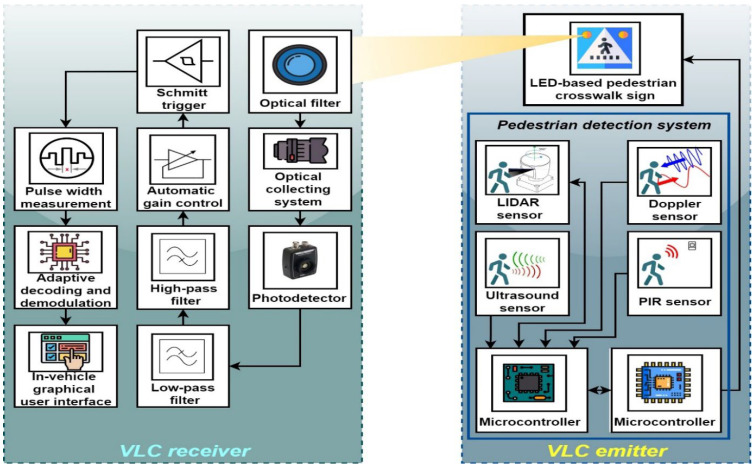
Schematic of the proposed system showing the interconnectivity between the VLC component and the pedestrian detection and localization component.

As shown in Figure 5, the two main components of the VLC system consist of a VLC emitter and a VLC receiver. The VLC emitter is developed based on an LED-based pedestrian crossing sign. It should be emphasized that the parameters of the LED-based pedestrian crosswalk sign are similar to the ones used on public roads, in terms of size, LED optical power, and height. 

The light intensity of the LED signaling lights, (i.e., *ON* or *OFF*) is controlled by the 180 MHz ARM Cortex M4 microcontroller through a digital power switch that commands the state of the LEDs. In line with the IEEE 802.15.7 standard (PHY I specification), the outdoor VLC system uses OOK modulation, Manchester codding, and a data rate of 100 kb/s. For simplicity and lower implementation cost, the system uses an asynchronous communication protocol. The asynchronous communication protocol includes a synchronization pattern that signals the VLC receiver that a new message is about to be received. Next, the PHY header of the frame provides the VLC receiver information concerning the selected modulation technique, the selected coding technique, the message data rate, and the message length. Based on this information, the VLC receiver is able to adjust its data extracting parameters and it can properly decode the information bits. Next, after the PHY header, the frame contains a start bit that marks the beginning of the data message, and a stop byte that marks the message end. The header of the message frame also provides the VLC receiver with information concerning the type of modulation, the coding technique, the data rate, and the length of the message. The parameters of the VLC emitter are summarized in Table 2. 

The VLC receiver is based on a structure developed by our research team, which is presented in detail in [27]. It consists of an optical collecting system, a signal processing component, and a data processing unit. In its turn, the optical collecting system integrates an optical filter that enhances the SNR by eliminating the unwanted optical spectrum component, an optical lens that increases the optical collecting area and in turn, the amount of light that reaches the photoelement’s surface, and a Thorlabs PDA100A2 optical detector based on a PIN photodiode connected in a transimpedance circuit. The signal processing block consists of a band-pass filter whose cut-off frequencies are determined based on the power spectral density of the 100 kHz Manchester encoded data, several amplification stages that are able to provide an overall amplification of up to 5000 times, a complementary automatic gain control stage that enables the VLC receiver to work at variable emitter–receiver distances and in mobile conditions, and a Schmitt trigger circuit that reestablish the signal’s square shape. Next, the signal is processed by the embedded 180 MHz ARM Cortex M4 microcontroller. This data processing unit is able to decode the binary data by using an efficient algorithm based on edge identification and pulse width measurement. The microcontroller is able to process the incoming data in real-time and it is also able to determine the Bit Error Ratio (BER) of the received data. In this case, the BER is only accomplished for the data bits, without including the start, and the stop bits in this evaluation. The parameters of the VLC receiver are summarized in Table 3.

## 4. Experimental Evaluation of the Pedestrian Crossing Assistance System

The following section presents the experimental evaluation of the proposed architecture. In order to have a wider view on the system performances, the testing procedure has been divided in two parts, one orientated on the accuracy of the pedestrian presence procedure, and the second one orientated toward the evaluation of the VLC data transmission system.

### 4.1. Experimental Testing of the Pedestrian Presence and Localization System

The first part of the experimental testing procedure was focused on the evaluation of the pedestrian presence system. After an initial calibration of the system in laboratory conditions, where the tests confirmed that the prototype is working as intended, a pedestrian crossing point has been recreated within the University of Suceava campus for outdoor tests.

**Figure 6:**
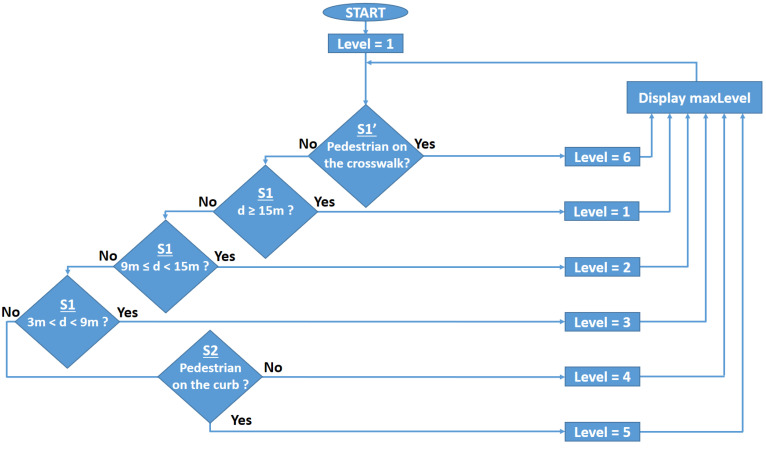
Workflow for the pedestrian presence and localization algorithm.

**Figure 7:**
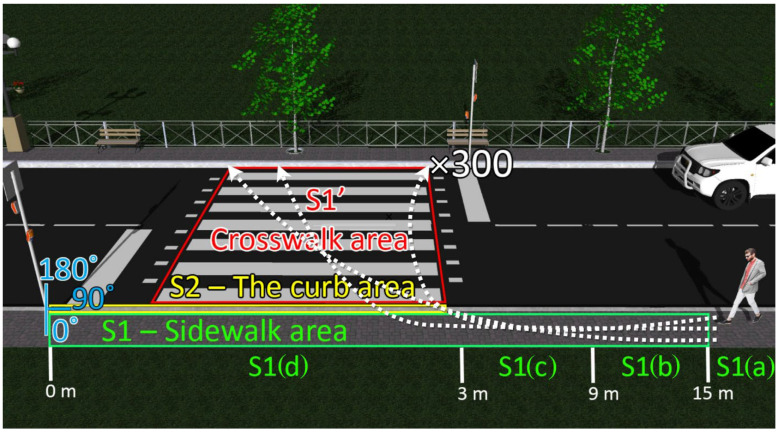
Schematic of the envisioned pedestrian detection component experimental setup: a volunteer, passes 300 times through the crosswalk area, crossing all zones. The software program monitors the detections for each zone and also, the overall pedestrian detections.

In the initial part of the outdoor experiments, the sensors of the pedestrian presence component were tested in the preparation for the main task. The conditions at the time were variable, with shady and sunny areas, on a summer day with temperatures between 20 and 25 °C in the shade, with solar irradiance between 2500 and 25,000 μW/cm^2^, and winds between 4 and 8 km/h. As expected, the PIR sensors did not perform well in these conditions, and even in shady areas, the false negative results were too many to consider the sensor reliable for the envisioned purpose. On the other hand, the microwave sensors were given a lot of false positive errors, due to the movement of leaves and branches of the surrounding trees, which also made this type of sensor unsuitable for this experiment. Instead, the ultrasound sensor was more reliable in the initial tests, and so, this was included in the workflow. For the LIDAR sensor, a more complex approach was needed for the initial tests, in order to also estimate not only the presence of a pedestrian but also the occupied zone. For this purpose, the LIDAR sensor was mounted on an angular controlled servo motor, and the measurements were made every second from 0° to 180° and vice versa in 2° steps. For the system calibration, the entire 180-degree zone was scanned a couple of times when no pedestrian was in the system’s range, and the distances measured for every step were averaged in order to obtain a scanned map of the area. After that, the prototype was started in the running mode, (i.e., continuous scanning), and based on the calibration values previously recorded, the software program whose workflow is illustrated in Figure 6, was able to pinpoint any presence in the covered zone when the distance measured for any angle was smaller than the calibration value. Once the calibration and the first tests confirmed that the system is running as intended, from the entire 180° scanned zone, the necessary limits were imposed for every step, in order to delineate only the zones of interest around the crosswalk, as it can be seen in Figure 7 and in Table 4. It should be mentioned here that one drawback was still encountered in this part of the experiment, namely the scanned part of the sunny area. In such conditions, the LIDAR was perturbed when it was facing these zones, so a better sensor should be considered to solve this issue in the future.

After all the preparations were made for the initial calibration and tests, the selected sensors, (i.e., ultrasound and LIDAR sensors) were activated in the workflow for the experimental evaluation of the zones and alarm levels already delineated. We chose to test the zones with only one pedestrian because his presence is harder to be detected by the sensors than a group of people.

The main part of this procedure was made in a shady part of the campus, where the irradiance was 2500–3000 μW/cm^2^, in order to avoid the blindness of the LIDAR sensor. As illustrated in Figure 7, three types of situations have been identified and tested:

- The S1’ area delineates the entire crosswalk, 3 m wide and 7 m long, covered by the LIDAR sensor scanning the zone from 90° to 180° in 2° steps. Any presence on the crosswalk will be detected by a lower measured distance than the limit imposed in the first part of the experiment, for every angle in this zone, and an alarm of Level 6 will be triggered;
- The S1 area delineates the sidewalk zone of interest, covered by the LIDAR sensor scanning the zone from 0° to 90° in 2° steps, further divided into four parts:
  (a) A more than 15 m zone from the sign, which will ensure that a pedestrian at a greater distance will not trigger a more than Level 1 alarm;
  (b) A 9 m to 15 m zone from the sign, which will ensure that a pedestrian in this zone will trigger a Level 2 alarm;
  (c) A 3 m to 9 m zone, which will ensure that a pedestrian in this zone will trigger a Level 3 alarm;
  (d) A 0 to 3 m zone, which will ensure that a pedestrian in this zone will trigger a Level 4 alarm.

A further zone could be delineated on the sidewalk in the opposite direction from the sign, in the 180° to 360° zone, but that would just replicate the tests made on this area. Nevertheless, the same divided zones can also be implemented, considering only the (a), (b), and (c), respectively, a more than 12 m zone, a 6 m to 12 m zone, and a 0 to 6 m zone from the sign.

- The S2 area delineates the curb zone of the sidewalk in front of the crosswalk area, covered by the LIDAR sensor and the ultrasound sensor, which will ensure that a pedestrian in this zone will trigger a Level 5 alarm, indicating that a person is on the verge of crossing the street.

As illustrated in Figure 7, the experimental tests cover 300 pedestrian passes for every zone, except for the Level 1 alarm, which should indicate the safest level, with a total of 1800 results. A brief description of the situations and a summary of the experimental results are summarized in Table 5. The experimental results resuming 300 pedestrian passes and 1800 tests show an overall 100% pedestrian presence accuracy. Nevertheless, these results also show that in one case, the detection rate gets down to 82.33%. Here, the errors are encountered for *Alert Level 2*, the lowest alert level, with the pedestrian located more than 9 m from the crosswalk. In this case, the errors are most probably generated by the fact that the LIDAR is scanning the surface at 2° resolution, which means that when the distance between LIDAR and pedestrians increases, the low scanning resolution introduces some errors. Nevertheless, the impact of these *Alert Level 2* errors is reduced as the system has an accuracy of at least 99% for *Alert Levels 3–6*. Furthermore, although these tests have been performed for only one sidewalk, the system is intended for a two sidewalks road, meaning that two LIDARs (i.e., one on each side of the road) will be performing the scanning, leading in turn to a significantly improved detection and localization accuracy. The experimental results also show that when an alarm level is triggered based on the data from more than one sensor, as in the case of *Alert Level 5*, an 100% pedestrian detection accuracy is achieved, emphasizing that high-performance PIR and Doppler sensors could be used to further improve pedestrian detection accuracy results. Anyway, one can see that the proposed design of the Pedestrian Presence and Localization System is very suitable for adequately locating the pedestrians. Even if for some particular area, the detection accuracy was lower than 100%, the overall detection rate is 100% since the pedestrian was detected in at least one area, and most important, detected with high accuracy in areas associated with *Alert Level 5* and *6.*

### 4.2. Experimental Testing of the Visible Light Communications System

**Figure 8:**
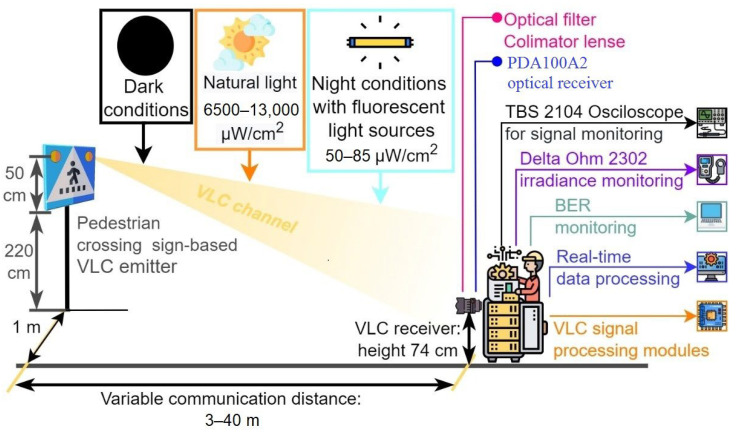
Schematic of the experimental setup.

**Figure 9:**
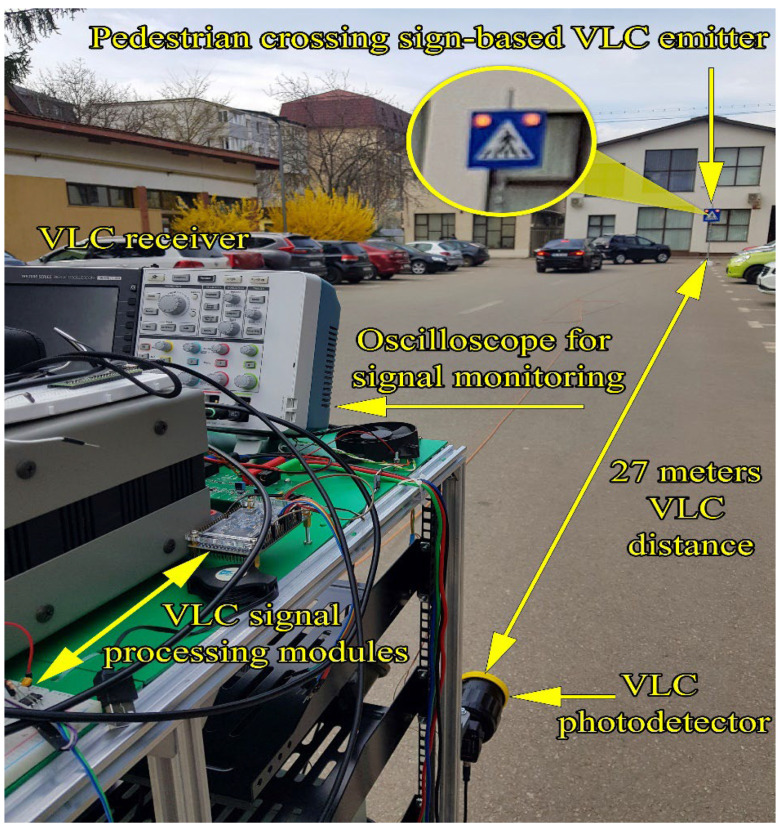
Visible light communications component outdoor experimental testing setup.

**Figure 10:**
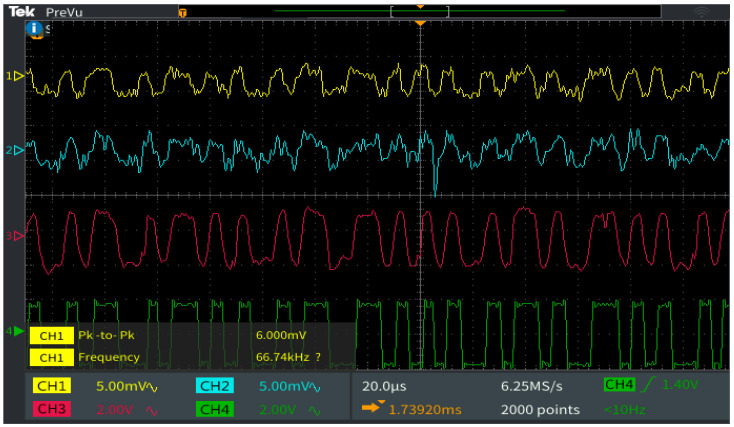
Oscilloscope screen showing the signal processing plan at the VLC receiver level: Channel 1 (yellow) shows the output of the optical receiver adjusted at 30 dB gain; Channel 2 (cyan) provides the output of the 1 kHz–200 kHz band-pass filter; Channel 3 (magenta) shows the amplified signal that will be fed into the Schmitt trigger circuit; Channel 4 (green) displays the final data signal that will be provided to the microcontroller for the data extraction process.

**Figure 11:**
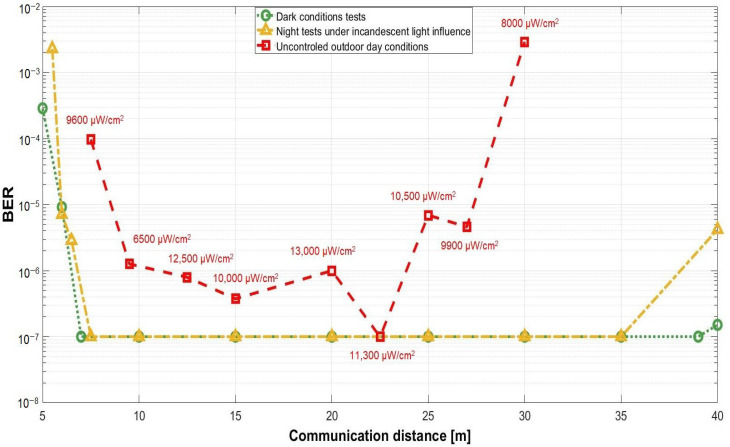
Bit error ratio results for the experimental evaluation of the visible light communications system in night conditions, under the influence of fluorescent lights, and in outdoor uncontrolled daytime conditions.

The next section of this article is focused on presenting the experiment testing procedure of the VLC component. These tests have been performed in laboratory nighttime darkness conditions, in laboratory nighttime conditions under direct exposure to fluorescent light sources having an irradiance at the VLC receiver level between 50 μW/cm^2^ and 85 μW/cm^2^, and in uncontrolled outdoor daytime conditions with sunlight irradiance at the VLC receiver level having values between 6500 μW/cm^2^ and 13,000 μW/cm^2^. The indoor night tests have been performed for a communication distance between 5 and 40 m, whereas the outdoor tests were for a distance between 6 and 27 m. As one can see, the testing scenarios replicate very well all the conditions to which the VLC system might be exposed in real-world working conditions. In line with the IEEE 802.15.7 standard, the VLC system used the OOK modulation and the Manchester coding. These tests have been performed at a data rate of 100 kb/s which is the highest data rate specified in the standard for PHY I outdoor applications using OOK modulation. In order to evaluate the raw performances of the VLC prototype, no forward error correcting codes have been used. During these tests, 10 million data packets have been transmitted, by cyclically repeating a 120 bits sequence. On the receiver side, the optical signal is electronically processed, and then, it is decoded by the 180 MHz microcontrollers in real time. Next, after the data are decoded, the microcontroller is also able to perform a BER analysis by comparing the received bits with the predefined bits sequence stored in the memory. The summary of parameters used for these tests is summarized in Table 6, whereas the experimental testing procedure is illustrated in Figure 8. Figure 9 shows the outdoor experimental setup, whereas Figure 10 illustrates the signal processing steps for a signal received from the VLC emitter for an I2V distance of 27 m in outdoor conditions. Next, the experimental results for the VLC component are presented in Figure 11 and discussed in detail in Section 5.1.

## 5. Discussion about the Importance of This Work, Differences from Other Approaches and Perspectives

### 5.1. Debate on the Results

As shown in Section 4.1, one can see that the proposed pedestrian detection architecture is successful in detecting pedestrians and in determining the street crossing action. As revealed by the experimental results, the architecture has an overall detection rate of 100%, meaning that the sensors detected the pedestrian every time in the 300 tests, which is the equivalent of a 95% confidence level. A lower detection accuracy of 82.33% has been achieved for the area associated with Alert Level 2, which is the case of a pedestrian being found at 9 to 15 m from the crosswalk. Nevertheless, as the accuracy for the other alert levels is of at least 99%, this should not affect the overall system performance. The tests have also shown a 100% pedestrian detection rate for the area associated with Alert Level 5, which was monitored by two sensors, (i.e., LIDAR and ultrasounds) and not only one. This indicates that if intended, the accuracy of the architecture could be further enhanced with the help of additional sensors that could provide backup and complementary support in pedestrian detection. Thus, the activation of each alert level could be determined based on the data received from more than one sensor. 

Nevertheless, as this article is mainly focused on using the VLC technology in solving a specific problem, (i.e., using a LED-based street crossing sign to broadcast whether or not a pedestrian is in the area), the pedestrian detection architecture is considered a proof-of-concept and not as a final system or as the central point of this work. Therefore, although it is highly efficient, this architecture can be further optimized to further improve the pedestrian detection ability, or it can be upgraded with artificial intelligence capabilities that could enable the system to predict the pedestrian trajectory and future intentions.

On the other hand, as shown in Section 4.2, the intensive testing of the VLC component has shown that the VLC technology is adequate in transmitting this information to the approaching vehicles, whereas it also showed some limitations. Nevertheless, before continuing with the discussion of the results, it should be remembered that these experimental tests have been performed in unfriendly circumstances generated by the conditions imposed by the real case scenario:

- Low power VLC emitter has a similar optical power as the ones encountered on standard roads, (i.e., 6.8 µW/cm^2^ at 1 m);
- A 2.7 m high traffic sign, similar to the height of a real traffic sign, which limits the coverage area and the irradiance level that reaches the VLC receiver;
- The VLC receiver is positioned at a height of only 74 cm–similar to the case when the VLC receiver is positioned at the vehicle front light level;
- Narrow FOV VLC receiver that enables enhanced noise resilience.

In the above-mentioned circumstances, the experimental results demonstrated a reliable communication range between 7 and 27 m in daytime conditions, being extended between 5 and 40 m in nighttime conditions. So, if it is to refer to the 27–40 m service area limit, one can see that this distance is sufficient to enable a vehicle running at 50 km/h to stop in full safety conditions. On the other hand, the 27–40 m maximum range is not as good compared to the distance provided in other vehicular VLC applications. For example, the V2V range can be extended up to 75 m when a vehicle taillight is used as a VLC emitter [27], and reach up to 185 m when a vehicle front lighting system is used as an emitter [24]. Extended communication ranges reaching 188 m can also be obtained in an I2V traffic light for vehicle applications [25]. Nevertheless, as mentioned above, the irradiance of the crosswalk LED sign is only 6.8 µW/cm^2^ at a 1 m distance with a 2.56 W LED power, which is significantly lower compared to the 9 W traffic light for example. Additionally, the 2.7 m height of the zebra crossing sign is also affecting the amount of received optical power at the VLC receiver level. 

Now, if it is to analyze these results, the analysis from the perspective of the car approaching the pedestrian crossing seems the most appropriate. So, as the vehicle is coming toward the pedestrian crosswalk, it will begin to receive the data concerning the state of the pedestrian crosswalk. This service area begins at about 30 m in daytime conditions and from about 40 m in nighttime conditions. It has to be emphasized here that due to the absence of interferences generated by sunlight, the systems performances are significantly better in nighttime conditions. This aspect is very important considering the fact that human sight is less efficient at night. Additionally, standard camera systems are also less performant in night conditions, meaning that the VLC solutions can help a human driver when a camera-based system would be less efficient. As expected, VLC system performances are lower at the far limit of the coverage area, with BER values reaching 10^−3^ values in daytime conditions and lower than 10^−5^ in nighttime conditions. The experimental results also show that the VLC receiver is able to properly filter the 100 Hz signal generated by the fluorescent lights, demonstrating that the system can properly work under the influence of the streetlights. Furthermore, as these results have been obtained without the use of FEC protocols, superior BER performances can be achieved with the integration of such error correcting protocols, further securing the system functionality in the 30–40 m region. Next, as the vehicle continues to move forward, the VLC distance is decreasing and the SNR is improving, enabling in turn BER around 10^−6^, even in daytime conditions. As the distance continues to decrease, the 2.7 m VLC emitter height corroborated with the ±20° VLC emitter’s angle, and the ±20° VLC receiver FOV affects the quantity of optical power being received, which, in turn, affects the SNR, explaining the BER increase in the close limit coverage area. On the other hand, in nighttime conditions, the absence of strong sunlight enables a higher SNR, and therefore, improved BER performances in the close limit coverage area.

Although for the purpose of these tests, a worst-case scenario was investigated, the performance can be improved by orienting the LED spot a few degrees toward the road/vehicle. In such a case, the irradiance of the optical signal received by the VLC receiver will increase, improving, in turn, the SNR and the BER, while also reducing the length of the initial no-coverage area. Additionally, on the receiver side, the performances could be further improved with the help of an adaptive FOV VLC receiver, and/or with the help of a VLC tracking system.

As discussed in the Introduction section, and as extensively debated in [14,15,16,17,18,19,20,21,22,23,27], the performances of vehicular VLC systems can be affected by the unpredictability of vehicle dynamics, and by weather conditions. With regard to the latter, there are two major kinds of phenomena. The first one is associated with situations in which intense sunlight can affect VLC receiver performances. With respect to this scenario, one can see that the VLC system has been tested in daytime conditions with sunlight irradiance at the VLC receiver level reaching 13,000 µW/cm^2^, which are above average sunlight irradiances. For this setup, the BER has been affected in the close limit and the far limit of the service area. Nevertheless, this BER increase was also caused by the limited VLC emitter–VLC receiver direct visibility. On the other hand, in the central region of the service area, (i.e., 10–25 m), the effect of the solar light did not decrease the BER below 10^−5^ limits, which proves the VLC receiver’s relatively high resilience to optical noise.

The second type of weather phenomena affecting VLC is the phenomena that affect light passage, (i.e., dust, fog, rain, snowfall, and blizzard). In this case, part of the data containing the light signal is prevented from reaching the VLC receiver, affecting in turn the SNR. On this topic, previous experience has shown that snowfall can increase the BER from 10^−7^–10^−6^ values up to 10^−5^ values, whereas snowfall associated with heavy blizzard further increases the BER at 10^−3^ values [21,22]. Now, in summary, both intense sunlight and weather phenomena that affect light passage depreciate the SNR. Nevertheless, these two types of phenomena are usually antagonistic, meaning that we cannot have intense sunlight and heavy fog or heavy snowfall at the same time. In such circumstances, one can conclude that current and previous experimental results indicate that in unfriendly conditions, the BER can increase up to 10^−5^–10^−3^.

### 5.2. Debate on the Importance of This Work

As far as we know, the proposed zebra crossing architecture is totally different from any other existing solutions that are aimed to improve the safety of pedestrians, as there is no similar work oriented toward this topic, based on the VLC technology. As the experimental results confirmed, the VLC technology is almost ready to make the transition from high potential technology to a viable solution suitable for improved road safety applications. The proposed prototype has demonstrated a 100% rate in pedestrian detection and a communication distance that can go up to 40 m while providing a 100 kb/s data rate. These results are very decent and highly suitable for improving vehicle awareness and pedestrian safety at the zebra crosswalk level. A previous attempt in using the VLC technology in pedestrian safety applications has been recently reported in [53]. Nevertheless, in this case, the optical camera visible light communication technology has been used to signal distracted pedestrians using their mobile phones that they are approaching a red traffic light signal. A different attempt at using the VLC technology in detecting hidden obstacles is found in [54]. In this work, potentially dangerous situations, such as a pedestrian crossing the street, are identified using an on-vehicle camera system. Next, this information is transmitted toward the vehicles behind, which are not able to detect the danger, using the taillights as a VLC transmitter. The following vehicle uses a camera system to receive this information using the concept of Optical Camera Communication (OCC) [55,56]. Although the working principle is remarkable, the system has several disadvantages generated by the limitations associated with OCC. So, the OCC technological data rate is limited to only a few kilobits per second, the communication distance is limited to a maximum of 20 m, and the latencies are unacceptably high, (i.e., 1 s in this case). Consequently, the performances of our proposed prototype are significantly superior in terms of communication distance, data rate, and latencies [23].

The problem of pedestrian crossing at the crosswalks levels has been addressed in [57]. In this case [57], the pedestrian detection is only managed with the help of PIR sensors whose performances might be affected in strong sunlight conditions, if additional sensors are not used for increased certainty. Additionally, different from the architecture proposed in the current article, the one proposed in [57] commands the switching of the traffic light color, without having the ability to transmit the data toward the approaching vehicles. The problem of using I2V systems to transmit such data to approaching vehicles has been addressed in [58], but only by simulation means.

Another important research direction oriented toward the detection of pedestrians by (autonomous) vehicles is based on the use of radar systems operating at various frequencies, between 24 and 300 GHz [59,60]. This approach is highly promising in detecting obstacles, but its performance might be significantly affected by mutual interferences once the technology is used on multiple vehicles [61]. Additionally, different from the proposed design, the approach based on radar systems is not able to detect pedestrians before they are already engaged in street crossing, nor they are capable to detect the pedestrians that are already on the zebra when the visibility is obstructed by other cars stopped in traffic on adjacent lanes. 

At the present time, most of the existing solutions focused on pedestrian presence are based on camera systems that use different video processing algorithms to detect pedestrians and estimate their future street crossing intention [62,63,64,65]. The probability of a pedestrian crossing or not is generally based on the analyses of the pedestrian’s posture and on the analysis of the posture changes in multiple frame images. The performances of such systems are currently being improved with the help of neural networks [66] or adversarial feature learning [67], which are orientated to establish the current pedestrian orientation [68] and the probability of future action [69]. Such systems can predict a future pedestrian’s street crossing intention with 0.6 s in advance while providing recognition accuracies higher than 93% [65]. Nevertheless, the performance of such solutions could be affected by the vehicle movement, involving in turn a constant rescaling process [70].

An interesting approach in identifying pedestrians is proposed in [71]. This solution is also based on cameras, but unlike most of the previously mentioned articles, which generally use on-vehicle camera systems, this one proposes the use of the traffic monitoring cameras. This solution has the advantage of being developed over an already implemented surveillance cameras infrastructure, offering the benefits of a dual use of the cameras and thus, of a lower cost.

In conclusion, based on the above-mentioned examples, one can see that the proposed architecture introduces a new approach in the design of smart pedestrian crossing points. Based on a multi-sensor fusion approach, the system is able to achieve a high pedestrian detection rate and establish a certain danger level. Then, based on the use of the VLC technology, the system is able to transmit this data toward the approaching vehicles, while ensuring a high confidence level imposed by communication-based vehicle safety applications. 

Another important contribution of this article comes from the fact that unlike many of the previous works, this one makes the transition from conceptual work to a practical application. So, this work seems to be the first case when the VLC technology is successfully being used to increase road safety at zebra pedestrian crossing points.

## 6. Conclusions

In the context where traffic accidents represent a major problem in contemporary society in general, and modern cities in particular, this article proposes a state-of-the-art technology used to address part of this issue. Thus, this article has proposed a new architecture aimed to assist drivers and/or autonomous vehicles in a safe manner for pedestrians’ street crossing. So, the proposed architecture aims to increase the protection factor for pedestrians, which are one of the most vulnerable road user categories, especially in urban areas.

The proposed architecture consists of a pedestrian detection and localization component that establishes the proximity of a pedestrian to the crosswalk, and a VLC component that transmits this information toward the approaching vehicles. The proposed architecture has been implemented at a real scale and has been tested in outdoor conditions similar to the ones encountered in practical situations. The experimental evaluation has shown an overall 100% successful detection rate, and a communication distance for the VLC component that reaches up to 27–40 m while providing a BER lower than 10^−5^. Therefore, the experimental results have confirmed the performance of the proposed architecture and its potential in ensuring enhanced protection for pedestrians at a street crossing.

In conclusion, this article has made an important transition from the point where the VLC technology is evaluated by simulation means or it is tested in laboratory conditions to the point where this highly potential technology is integrated into a real application and it is solving a problem. This aspect is highly important as it demonstrates the real progress of the technology and its potential in being almost ready for deployment in practice.

## Figures and Tables

**Table 1:** Summary of the sensors tested for pedestrian detection, sensors characteristics, and sensors purposes.

Sensor Type	Sensor Model	Sensor Detection Range	Sensor Detection Angle	Sensor Purpose
Lidar	Lite V3	40 m	±0.25°	Detection of pedestrians in the area of interest, including the sidewalk area
Ultrasonic	HC-SR04	4 m	±7.5°	Detection of pedestrians at the intersection between the sidewalk and the crosswalk
Passive Infra-Red	HC-SR501	6 m	±60°	Detection of pedestrians in the crosswalk vicinity and on the crosswalk
Microwaves	RCWL-0516	6 m	±85°	Detection of pedestrians in the crosswalk vicinity and on the crosswalk

**Table 2:** Visible light communications emitter parameters.

VLC Emitter Parameter	Feature/Measure
VLC emitter type	LED-based pedestrian crossing traffic sign (60 × 60 cm)
Total LED power	1.28 W for a single side LEDs group 2.56 W for both LED groups
VLC emitter optical irradiance (measured at 1 m distance)	4.8 µW/cm^2^ for a single side LEDs group 6.8 µW/cm^2^ for both LED groups
VLC emitter half angle	20°
VLC emitter center wavelength	610 ± 20 nm
VLC emitter height	270 cm (similar to the real case)
Modulation technique	OOK
Coding technique	Manchester
Data rate	100 kb/s
Communication type	Asynchronous communication protocol

**Table 3:** Visible light communications receiver parameters.

VLC Receiver Parameter	Feature/Measure
VLC receiver type	PIN photodiode based Thorlabs PDA100A2 optical detector
VLC receiver optical filter characteristics	640 ± 45 nm
VLC receiver optical system characteristics	2-inch optical lens
VLC receiver FOV	±20°
VLC receiver height	74 cm (similar to the real case when the VLC receiver is mounted at the vehicle front lights level)
VLC receiver data processing unit	180 MHz ARM Cortex M4 microcontroller
VLC receiver capabilities	Real-time data processing having data rates up to 500 kb/s and real-time bit error ratio processing

**Table 4:** Table defining the alert level concerning the occupied areas with pedestrians near the crosswalk.

Alert Level	Pedestrian Action	Scenario Description	Active Sensors/Sensors Regions		
			LIDAR		Ultrasound
			S1	S1’	S2
1 (lowest)	No pedestrian in the area of interest	The sensors did not detect any presence in the supervised area	d ˃ 15 m	0	0
2	Pedestrian in the area with no evidence concerning any intention of using the crosswalk	At least one person has been identified in the area, but at this point the person’s location is more than 9 m away from the crosswalk	9 m ≤ d ≤ 15 m	0	0
3	Pedestrian in the area with low evidence concerning the intention of using the crosswalk	A person has been identified by the sensors, and at this point, the person is near the crosswalk.	3 m ≤ d ≤ 9 m	0	0
4	Pedestrian in the area with high evidence concerning the intention of using the crosswalk	A person has been identified by sensors, indicating that there is a real possibility that the person will use the crosswalk.	d ≤ 3 m	0	0
5	Pedestrian beginning to use the crosswalk	The sensors have identified a person in the security region, indicating that the pedestrian is on the verge of crossing the street	d ≤ 3 m and 86° < angle < 90°	0	d ≤ 4 m
6 (highest)	Pedestrian on the crosswalk	The sensors have identified a person on the crosswalk. At this point, it is sure that the person is crossing the street.	X	1	X

S1—Sidewalk area; S1’—crosswalk area; S2—the curb area in the vicinity of the crosswalk; 0—the sensors are not detecting anything in that area; 1—the sensors are detecting something in that area; X—the states of the sensors are not relevant in this situation.

**Table 5:** Summary of the Pedestrian Presence and Localization System experimental evaluation.

Testing Scenario	Description of the Scenario	Total Number of Tests	True Positive	False Positive	False Negative	Overall Rate (True Positive/Total No. of Tests)
Alert Level 2 (lowest)	Pedestrian detected in the area associated with *Alert Level 2*	300	247	0	53	82.33%
Alert Level 3	Pedestrian detected in the area associated with *Alert Level 3*	300	297	0	3	99%
Alert Level 4	Pedestrian detected in the area associated with *Alert Level 4*	300	297	0	3	99%
Alert Level 5	Pedestrian detected in the area associated with *Alert Level 5*	300	300	0	0	100%
Alert Level 6 (highest)	Pedestrian detected in the area associated with *Alert Level 6*	300	299	0	1	99.66%
Pedestrian detected	Pedestrian detected in *at least one area*	300	300	0	0	100%

**Table 6:** Summary of the experimental parameters.

Parameter	Feature/Value
VLC Emitter type	LED-based standard pedestrian crossing traffic sign
VLC Emitter height	270 cm
VLC emitter optical irradiance	6.8 μW/cm^2^ at 1 m distance
VLC emitter wavelength	610 nm ± 20 nm
Receiver type	±15° PIN photodiode-based receiver
Receiver height	74 cm
Received signal power	6.8 μW/cm^2^–0.005 μW/cm^2^
Noise irradiance induced by the natural light	6500–13,000 μW/cm^2^
Noise irradiance induced by fluorescent lights	50–85 μW/cm^2^
Emitter–receiver distance	3–40 m
Emitter–receiver lateral distance	1 m
Modulations, coding techniques and data rates	OOK modulation with Manchester coding at 100 kb/s data rate
Number of bits in a data set	10 million
Testing conditions	Nighttime: in darkness Nighttime: under the influence of fluorescent light sources Daytime: under sunlight exposure

## Data Availability

Not applicable.
